# *Hypericum perforatum*: Traditional uses, clinical trials, and drug interactions

**DOI:** 10.22038/IJBMS.2022.65112.14338

**Published:** 2022-09

**Authors:** Seyedeh Zahra Nobakht, Maryam Akaberi, Amir Hooshang Mohammadpour, Ali Tafazoli Moghadam, Seyed Ahmad Emami

**Affiliations:** 1 Department of Traditional Pharmacy, School of Pharmacy, Mashhad University of Medical Sciences, Mashhad, Iran; 2 Department of Pharmacognosy, School of Pharmacy, Mashhad University of Medical Sciences, Mashhad, Iran; 3 Department of Clinical Pharmacy, School of Pharmacy, Mashhad University of Medical Sciences, Mashhad, Iran; 4 Department of Clinical Pharmacy, School of Pharmacy, Shahid Beheshti University of Medical Sciences, Tehran, Iran

**Keywords:** Clinical trials, Hyperforin, Hypericaceae, Hypericin, Hypericum perforatum, St. John’s wort

## Abstract

*Hypericum perforatum* (Hypericaceae), known as Saint John’s wort (SJW), has been used in different systems of traditional medicine such as Chinese traditional medicine, Greek traditional medicine, and Islamic traditional medicine. The plant and its active constituents such as hyperforin and hypericin have a wide range of medicinal uses, particularly as anti-depressant, wound-healing, and antibacterial agents. In recent decades, many clinical trials have been performed to investigate the safety and efficacy of this medicinal plant. However, to the best on our knowledge, there is no comprehensive review article in this regard. In the current study, we aim to have a comprehensive review of the clinical trials of SJW to evaluate its efficacy and safety as well as its application in traditional medicine. Clinical studies investigating the safety, interactions, and efficacy of SJW were identified and summarized, including contributions from 2000 until December 2021. According to the results, these clinical studies were divided into three main categories based on the type of disease: psychiatric, endocrine, and skin problems. Important details of the studies, including the type and duration of the study, the type and percentage of the effective compounds or the extract used, the number of patients, and the obtained results were also discussed. In addition, co-administration and drug interaction of SJW with other drugs were summarized. SJW is a valuable medicinal plant, especially for psychiatric disorders. However, precautions should be taken while administrating the plant.

## Introduction

The *Hypericum* Tourn. ex L. is a cosmopolitan genus with 508 species worldwide. *Hypericum dogonbadanicum* Assadi is the only endemic species of the genus in Iran (1). The most well-known species of this genus is *Hypericum perforatum *L. (Hypericaceae), known as St. John’s wort (SJW). It is an herbaceous perennial plant native to western Asia, Europe, and northern Africa (2). The plant has been given its common name due to blooming in midsummer around St. John the Baptistʼs day in June. Klamath weed, Tipton’s weed, Rosin-rose, Goat weed, etc., are the less common names (3).

It is a very popular traditional herbal medicine due to its wide range of applications, including treatment of skin problems such as wound healing in burns, stomach ulcers, biliary disorders, inflammation of the bronchi and genitourinary system, colds, migraines, headache, diabetes mellitus, and obesity (4, 5). However, the reason for its popularity can be attributed to the effectiveness of this plant in the treatment of mild to moderate depression (6).

Because of its wide therapeutic effects, many clinical trials have evaluated the efficacy and safety of SJW. Since there is not a recent review on the clinical trials studying the efficacy and safety of SJW, we aim to have a comprehensive review of the clinical trials studying the interactions, safety, and efficacy of SJW and its related compounds and preparations in the present study.

***Traditional medicine***

*Hypericum perforatum* has been widely used in different systems of traditional medicine including traditional Chinese medicine, Islamic medicine, and Greek medicine.

*Traditional Chinese medicine (TCM)*

In this traditional system of medicine, SJW is known as Guan Ye Lian Qiao. Names including, Xiao Zhong Huang, and Xiao Dui Yue Cao (Guizhou), Guo Lu Huang Gan, Shan Bian, Qian Ceng Lou, Shang Tian Ti (Sichuan), Shan Han Lin Cao (Jiangsu), Da Dui Ye Cao (Hubei), Xiao ye jin si tao (Henan), and xiao Liu Ji Nu (Shaanxi) are its aliases. Ethnobotanical studies show that this plant has been used for hematemesis, hemoptysis metrorrhagia, irregular menstruation, traumatic hemorrhage, jaundice, acute mastitis, sore throat, urinary tract infection, swelling and pain of the eye, sore furuncle, burn, rheumatic arthritis, and wounds and bruises in TCM (7). SJW has not been used as an antidepressant in TCM although it has a long reputation for this purpose in Europe. According to traditional textbooks, the plant has a bitter and astringent taste, and a neutral nature (8). 

*Greek medicine*

Ancient Greek physicians such as Dioscorides, Theophrastus, and Galen used SJW to treat diseases such as snake or reptile bites, gastrointestinal distress, menstrual cramping, melancholy, depression, ulcers, superficial wounds, burns, and sciatica (9). SJW oil made with flowering tops of this plant was also used by surgeons to disinfect wounds and also heal bruises (10).

*Islamic traditional medicine*

Well-known physicians of Islamic traditional medicine**,** such as Avicenna, Râzi (Rhazes), Anṭâki, Herawi, Ansâri Shirazi, Ghassâni, Ibn Beyṭâr, and ʿAqili have mentioned various therapeutic applications for SJW. According to the contents written in the reference books of Islamic traditional medicine, the poultice of SJW has been used to heal infectious wounds, burns, and bruises (11-16). Laxative effects for the seeds of SJW have also been mentioned in several references (11, 12, 16). Other uses for this plant include improving sciatica (11, 12, 15-18). Ibn Sinâ (Avicenna) prepared a decoction of this plant in wine and prescribed it for forty consecutive days to alleviate this disease (13). Also, topical application of its combined poultice with olive oil has been used in the treatment of paralysis with the curvature of the back of the neck (16, 19). In general, other common uses of SJW in Islamic traditional medicine include diuretic, emmenagogues, antipyretic (especially malarial fever), antispasmodic, anti-gout, and anti-hemorrhoidal effects (11, 14-17, 19). In addition to the previous cases, SJW has been used in the treatment of jaundice, polydipsia, severe swelling, urinary stones, and even induction of abortion (11-13, 19).

***Phytochemical constituents***

**Figure 1:**
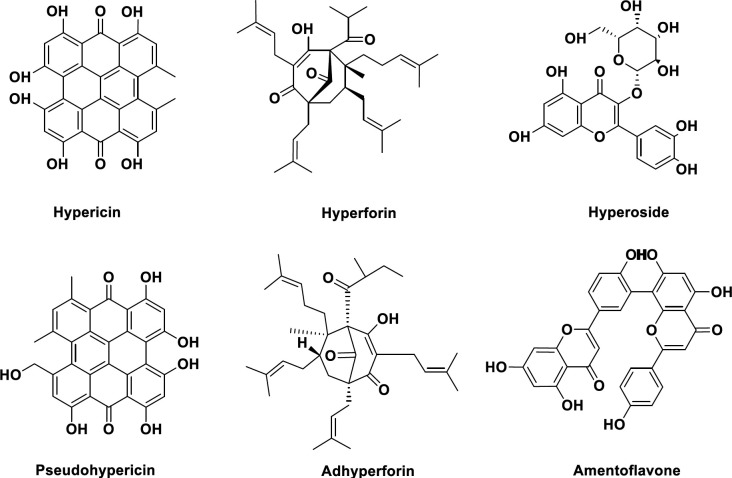
Important biologically active constituents of *Hypericum perforatum*

*Hypericum perforatum* contains a wide range of chemical compounds including volatile oils, flavonoids, anthraquinone derivatives (such as naphthodianthrones), prenylated phloroglucinols, tannins, xanthones, and other miscellaneous compounds. However, the therapeutical important compounds in *Hypericum* species include phloroglucinols including hyperforin, naphthodianthrones including hypericin and pseudohypericin, and flavonoids such as quercetin, quercitrin, rutin, and hyperoside (20). The standardization of SJW is normally based on hypericin and hyperforin contents (Figure 1) (21).

*Hypericum perforatum*

***Pharmacological effects of hypericum perforatum***

A large number of *in vitro* and *in vivo* studies have investigated the therapeutic effects of SJW and its constituents. In the following paragraphs, we will briefly discuss their therapeutic effects.

***Anti-depressant effects***

Hypericin was introduced as one of the main possible active compounds. Inhibiting the monoamine oxidase enzyme is a possible mechanism of action for hypericin (22). SJW is also able to inhibit the reabsorption of dopamine, serotonin, noradrenaline, L-glutamate, and γ-aminobutyric acid in nerve terminals (23). Moreover, several flavonoids such as quercetin, luteolin, and kaempferol have shown anti-depressant effects (24).

***Analgesic effects***

In a review study on the therapeutic effects of SJW, low doses of dry extract of the plant exhibited analgesic effects and strengthened the effect of opioids in acute and chronic animal pain models. *In vitro* and *in vivo* studies show that the compounds hypericin and hyperforin are responsible for such effects (25).

***Metabolic syndrome improving effects***

In the diet-induced obesity and metabolic syndrome animal model of study, administration of SJW extract could improve glucose and fat metabolism and insulin resistance (26). In addition, in a hyperlipidemia animal model of study, the extract of this plant could reduce LDL-CH and total cholesterol without affecting triglycerides and HDL-CH, improve liver parameters and decrease oxidative damage including malondialdehyde, aspartate aminotransferase, and alanine aminotransferase (27).

***Antimicrobial effects***

Antimicrobial effects have also been reported for the constituents of this plant. For instance, hyperforin has shown significant antimicrobial effects against *Staphylococcus aureus* strains (28). The plant has exerted not only antibacteria but also anti-fungal and anti-yeast activities. Aromatic polyketides such as hypericin have shown activities against pathogenic fungi and yeasts such as* Trichophyton rubrum, Fusarium oxysporum, Microsporum canis, Pichia fermentans*, *Exophiala dermatitidis, Kluyveromyces marxianus, Candida albicans, *and *Saccharomyces cerevisiae *(29). Moreover, studies have reported antiparasitic effects for hypericin and hyperforin against malaria and leishmaniosis parasites (30). Hypericin is also effective against viral protease that is well known for its activity against several viruses namely, herpes simplex, bronchitis, influenza A, and human immune (31, 32).

***Antineoplastic effects***

Hypericin is reported to have remarkable anti-neoplastic effects among all the compounds isolated from SJW. Recently, it has been applied as a phototherapy drug helping to treat cancer (33). Sensitivity to light from hypericin mainly affects the mitochondria or endoplasmic reticulum-Golgi complex leading to cell apoptosis (34). Hypericin has been shown to have activities against a range of cell lines including melanoma and breast cancerous cells. Exfoliation of phosphatidylserines, cell shrinkage, loss of cell membrane integrity, and caspase-dependent, as well as independent apoptotic modes, are some mechanisms of action (35). In addition, hyperoside, another phytochemical constituent from this plant has shown to have inhibitory effects against cancerous cell lines by inducing apoptosis and repressing cell proliferation (36).

***Wound healing effects***

**Figure 2:**
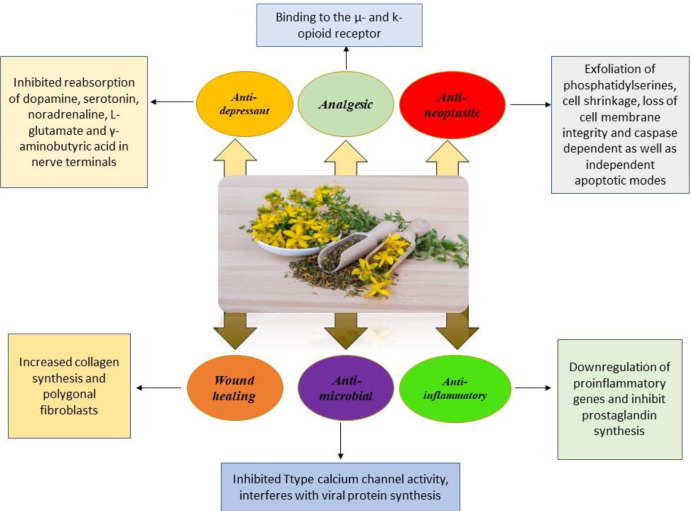
The pharmacological effects of *Hypericum perforatum*

In a review of topical products containing plants, researchers have suggested SJW products containing oil and tincture of the plant for the treatment of mild wounds, burns, sunburn, scratches, bruises, heat burns, fire, muscle aches, and other problems (37). Synergistic effects of hypericin, isoquercitrin, rutin,hyperoside, and epicatechin may cause wound healing effects of this plant (37, 38). *In vitro* studies show that the possible mechanism of wound healing is by increasing in production and activation of fibroblast collagen cells (39). The mechanism of pharmacological effects of SJW is summarized in Figure 2.

## Methods

The scientific databases including Scopus, Web of Science, and PubMed were searched to access all relevant books and papers in English until 2021. The keywords were “*Hypericum perforatum”* OR “St John’s wort” AND “clinical trial” OR “clinical study”. All English relevant papers from 2000 to 2021 were included. 

## Clinical Aspects and Safety of SJW

In this section, we will discuss the clinical studies investigating the efficacy and safety of SJW as well as its major constituents.

***Psychiatric disorders***

Most of the reviewed clinical trials (2000 to 2021) are devoted to depressive disorders and their types (Table 1). However, many other neurological and psychiatric problems such as insomnia, fatigue, obsessive-compulsive disorder, attention-deficit hyperactivity disorder, autistic disorder, social anxiety disorder, nervous agitation, short-term memory, and somatoform disorders have also been investigated. In patients suffering from depression, SJW has been found to be able to improve CGI and reduce HAM-D scores, relapse rate, and adverse effects significantly compared with placebo and other anti-depressant drugs (40, 41). The results of the studies on social phobia and polyneuropathy failed to provide evidence for the efficacy of SJW (42, 43). Opposite results have been observed in clinical trials conducted on OCD. In a clinical study, SJW improved the patient’s condition by increasing Y-BOCS scores, but in another study, it did not have a significant effect on the Y‐BOCS score (44, 45). Different study designs and different formulations used might explain these opposite findings. These opposite results were also observed in patients with attention definition disorder (46, 47). In a long-term safety study on 440 patients with mild to moderate depression, a relatively high safety of this plant was observed (48). In two studies, Chinese herbal remedies, mainly SJW and *Acanthopanax*, showed significant effects in reducing depressive symptoms and improving post-stroke motor symptoms (49, 50). It is suggested that more extensive studies be performed with higher doses of SJW or in combination with other medicinal plants affecting psychiatric disorders such as *Valeriana officinalis* and *Lavandula angustifolia* to perhaps achieve more effective results. Also, in the case of studies performed on severe depression, the patient should be monitored for physical harm and suicide attempt. It is also recommended to use standard extracts for studies (Table 1).

***Endocrine disorders***

Studies show that SJW and its constituents have promising activities against endocrine disorders. According to Table 2, PMS and menopausal disorders account for the largest number of clinical trials of SJW in endocrine disorders. These studies show positive results in improving the symptoms of hot flashes and the mood and behavioral symptoms associated with these disorders (64-67). However, SJW has not been as successful in relieving the pain of PMS as it is in improving its psychological symptoms (65). Another clinical trial has been performed on polycystic ovary syndrome and examined the simultaneous effect of lifestyle and consumption of several herbal compounds, including *Hypericum*. The results of this study showed that the combination was useful in improving blood pressure, BMI, insulin resistance, and psychological problems of PCOS (68). There have also been limited studies on the effect of *Hypericum* on the concentrations of steroid hormones such as ACTH, cortisol, and prolactin, which require further clinical studies in the future to ensure the results (69, 70).

***Skin diseases***

According to the results of Table 3, topical forms of SJW have shown beneficial effects in reducing inflammation, itching, and redness of scars in cesarean section and episiotomy (75, 76). Also compared with acyclovir, SJW has caused a better reduction in burning sensation and parameters of acute pain, erythema, and vesiculation in HSV-1 and HSV-2 lesions (77). Reducing TNF-α concentrations in the dermis, endothelial, and dendrite cells in patients with plaque-type psoriasis can be one of the mechanisms of this plant in healing skin lesions (78, 79). But it seems that the analgesic effects of this drug have not been enough for the burning mouth syndrome (80). In other clinical trials on non-melanoma skin cancers, the complete clinical response has been 50% for AKs, 28% in superficial BCC patients, and 40% in patients suffering from Bowen’s disease (81). The use of modern methods of drug delivery to the skin for higher penetration of the active ingredient in topical formulations and appropriate standardization based on the active ingredients of the drug can produce better results.

***Co-administration of SJW with other drugs***

According to Table 4, clinical trials performed with concomitant administration of SJW with other drugs are generally divided into four categories based on the mechanism of interaction:

First category: One of the most important features of SJW is its inductive effects on P-glycoprotein (P-gp) and hepatic cytochrome P450 enzymes including CYP3A4, CYP2C19, CYP2C9, CYP1A2, and CYP2D6 (82). Hyperforin plays an important role in the induction of CYP enzymes and P-gp by activating the pregnane X receptor (PXR) (83). Induction of these enzymes can reduce the concentration of drugs that are metabolized by these cytochromes or increase the effect of drugs such as clopidogrel that are converted to the active form by these enzymes (84). Clinical trials in this category investigate drug interactions in the concentration and metabolism of drugs with SJW. According to the data in this table, when using low-dose hyperforin products such as Ze 117, the use of SJW could not have a significant effect on the pharmacokinetics of drugs (85-87). However, extracts containing high doses of hyperforin, such as Jarsin® and Movina^®^, have significantly increased clearance, decreased the concentration and effectiveness of drugs, and sometimes failed treatment (88-92). Drugs that are affected by induction of CYP 450 with SJW include bupropion, oral contraceptives, docetaxel, rifampicin, rivaroxaban, oxycodone, oral s-ketamine, cyclosporine, tacrolimus, atorvastatin, irinotecan, zolpidem, metformin, and simvastatin (88, 93-105).

Second category: The activity of liver CYP450 enzymes in humans is genetically different and people are divided into three genotypes: extensive, poor, and ultra-rapid metabolizers (89). Studies in this category include SJW interactions with drugs in different genotypes. According to the results of the table, those who are rapid and extensive metabolizers are more affected by the inductive effects of SJW and show more drug interactions with this plant (89, 106, 107).

Third category: Evaluation of efficacy and safety of SJW compared SSRIs drugs is another group of clinical trials in this table. Increased Hamilton depression total score and rates of remission in the SJW group were better than paroxetine and fluoxetine in people with MDD. In addition, the side effects of SJW were less reported than with these drugs (108, 109).

Fourth category: The latest group of clinical studies in this table discusses the concomitant use of SJW with a potent CYP450 inhibitor and its effect on drug concentrations. Studies show that the use of ritonavir and ketoconazole in combination with SJW increases the concentration of midazolam and decreases its clearance, indicating the superiority of the inhibitory effects of these drugs over SJW induction. Of course, these results depend on the amount of hyperforin in the extracts used (110, 111). In the following paragraphs, the drugs interactions of SJW are discussed in detail:

*Contraindicated interactions*

*Irinotecan*

Avoid consuming SJW with irinotecan at the same time. The effect of SJW on the metabolizing enzymes of irinotecan may continue for several weeks after stopping the consumption of SJW. Therefore, if the patient is being treated with irinotecan and SJW, he should stop consuming SJW and, if possible, use the drug irinotecan with a delay and after 2 weeks (101).

*Major risk interactions*

*Antiviral drugs*

Drug resistance and treatment failure are two of the most important risks that can occur with the concomitant use of SJW and antiviral drugs such as indinavir, which is a viral protease inhibitor (112). Because other protease inhibitors, such as ritonavir and saquinavir, are also metabolized by liver cytochromes, SJW can also reduce their plasma concentrations and effectiveness (113, 114). Therefore, concomitant use of these drugs should be avoided.

*Chemotherapeutic drugs*

Docetaxel and imatinib are metabolized by hepatic cytochromes such as CYP3A. Their plasma concentrations and efficacy could be decreased when co-administered with SJW leading to treatment failure in cancer patients (95, 115).

*Immunosuppressant*

One of the most important drug interactions observed with SJW is related to the simultaneous use of this plant and drugs used after transplantation such as cyclosporine and tacrolimus, which reduces their plasma concentrations (85, 94, 100). Disruption of appropriate doses of these drugs has reportedly led to organ transplant rejection in several transplant recipients or put them at risk for transplant rejection (85, 116, 117).

*Warfarin and digoxin*

 Another very important interaction that should be considered by physicians when prescribing this drug is the concomitant administration of SJW with drugs such as digoxin and warfarin that have a narrow therapeutic index (118, 119). In a clinical trial, SJW reduced the therapeutic concentration of warfarin by increasing its clearance which increased the risk of blood clots (120). In a study on the simultaneous administration of SJW with digoxin, researchers found that only extracts containing high doses of hyperforin could have a significant effect on digoxin concentration, but more careful studies are needed to determine the possible amount of hyperforin to prevent possible side effects of digoxin dose changes (87).

*Moderate risk interactions*

*Metformin*

Due to the increase in glucose tolerance with increased insulin secretion independent of insulin sensitivity, it is recommended to monitor the symptoms of hypoglycemia in concomitant use of hypoglycemic drugs such as metformin with SJW (102).

*Oral contraceptives*

SJW can reduce the half-life of norethindrone and ethinyl estradiol by inducing CYP3A and their metabolism (96). It could increase the chance of ovulation and breakthrough bleeding and induction of unwanted pregnancy (96, 103). Therefore, co-administration of SJW with contraceptives is not recommended in women who do not intend to become pregnant at all.

*Statins*

Several clinical studies have reported that the effectiveness of lipid-lowering drugs such as atorvastatin, simvastatin, and rosuvastatin in concomitant use with SJW could be decreased. Increased total cholesterol and LDL cholesterol are among the proposed mechanisms for SJW interactions with statins (88, 100). In general, it is recommended that patients should avoid consuming SJW with blood lipid-lowering drugs at the same time.

*Oxycodone*

The metabolism of oxycodone can be increased while co-administered with SJW leading to the reduced analgesic effects of oxycodone. Thus, simultaneous administration of these two drugs should be dose adjusted (97).

*Rifampicin*

Based on the clinical study performed on the simultaneous administration of rifampicin and SJW, photosensitivity was observed only in the study group of women. Therefore, in prescribing SJW with rifampicin, the aggravation of side effects of photosensitivity caused by hypericin should be considered (98).

*Zolpidem*

Induction of CYP3A by SJW reduces the plasma concentration of zolpidem (105). Therefore, co-administration of this drug with tea grass also requires dose adjustment.

**Table 1:** Clinical trials of St. John’s wort on psychiatric disorders

**Disease**	**Extract/Compd.**	**Dosing & Duration**	**Study design & sample size**	**Result**	**Ref.**
ADHD	SJW extract	30 mg QD; 4 weeks	An open trial; three 14-16-year-old male psychiatric patients	↑ Patients' mean scores for Conners' hyperactivity inattention	(46)
ADHD	A 0.3% hypericin standardized extract	300 mg TID; 8 weeks	A double-blind RCT; 54 children (6-17 years) with ADHA	No significant change in ADHD Rating Scale-IV scores between the treatment and placebo groups	(47)
Atypical depression	LI160^1^	300 mg BID; 8-week	A double-blind RCT; 200 patients with mild to moderate severity of major depression (18-70 years)	↓ HAM-D17 for LI160 compared with placebo significantly Beneficial effect of LI160 in atypical depression	(51)
Autistic disorder	SJW extract	20 mg QD; 4 weeks	An open trial; 2 male patients (19.4–22.4 years with autistic disorder (IQs ranged from 58 to 72)	↓ Irritability in the short term ↑ Stereotypy and inappropriate speech factors slightly No significant improvement in clinical ratings (Anger and Speech Deviance factors, Psychiatric Rating Scale Autism, Global Assessment Scale, and Clinical Global Impressions efficacy)	(52)
Depression	IperiPlex^®^^2 ^and Nervaxon^®^^3^	300 mg BID; 12 months	A retrospective, 12-month, open-label, observational, controlled trial; 60 patients with moderate depression (18–75 years)	Twice the effect of IperiPlex® over Nervaxon® Failure to observe the therapeutic effects of Nervaxon^®^ for six months and its effectiveness after 12 demonstrated. Significant effectiveness of IperiPlex® in both 6- and 12-month groups	(53)
Depression	WS® 5570	(600-1200) mg QD; 6 weeks	A double-blind, multi-center RCT; 332 patients with mild or moderate depression (18–65 years)	↓ HAM-D total scores significantly Being safe and more effective than placebo	(40)
Depressive syndrome	Helarium-425®^4^	One capsule QD; 12 weeks	An open study; 1778 patients with depressive disorders (18–97 years)	↑ CGI scale (from 21.6% at the admission to 72.4%) ↓ Incidence of adverse drug reactions	(41)
Fatigue	Kira^®^	Tablet Kira TDS; 6 weeks	A pilot uncontrolled, open study; 20 patients with complaints of fatigue (32-60 years)	↓ Symptoms of fatigue	(54)
Insomnia	Kira^®^^5^	0.9 mg (in 11 subjects) and 1.8 mg (in 10 subjects); 1-2 weeks	A double-blind, placebo-controlled, balanced order, cross-over study; 21 healthy subjects	↑ Latency to REM sleep without any other effect on sleep architecture in both doses	(55)
Major depressive disorder	LI-160	900 mg QD; 26 weeks	A double-blind RCT; 124 patients with major depressive disorder (mean age of the group: 46 ± 13.0 for sertraline, 42 ± 13.0 for placebo and 45 ± 12.3 for SJW)	Both SJW extract and sertraline were therapeutically effective compared with the placebo	(56)
Major depressive disorder	LI 160	300 mg TID; 12 weeks	An active parallel-group double-blind RCT; 39 patients with major depressive disorder (18–65 years)	Further reduction of HDRS-17 scores in patients with early response to clinical improvement (occurring during the first 2 weeks) compared with patients with delayed onset of clinical recovery	(57)
Mild to moderate depression	Ze 117	500 mg extract QD; 1 year	A long-term safety study; 440 patients with mild to moderate depression (up to 18 years)	↓ Mean HAM-D scores and CGI scores No age-related difference in the safety of the applied medication No change in BMI No changes in clinical chemistry and electrocardiogram recordings	(48)
Moderate depression	WS® 5570 or Paroxetine (20 mg/d)	300 mg TID; 6 weeks	A double-blind, double-dummy, randomized phase III trial; 64 patients with moderate depression (18–70 years)	↓ HAM-D total score significantly in WS® 5570 group compared with paroxetine group	(58)
Nervous agitation	Combination of 3 dry extracts^5^	1–3 tablets QD; 4 weeks	A multicenter, prospective observational study; 115 children with social withdrawal, attention problems, and/or anxious/depressive (6–12 years).	↓ Attention problems, social withdrawal, and anxious/depressive	(59)
OCD	Extract standardized to 0.3% hypericin	450 mg BID; 12 weeks	An open-label trial; 12 patients with OCD	↑ Y-BOCS	(45)
OCD	LI 160	Flexible dose (600–1800 mg QD); 12 weeks	A double‐blind placebo-controlled trial; 60 patients with OCD (18–65 years)	No significant difference between the mean change on the Y‐BOCS score with SJW and placebo	(44)
Polyneuropathy	Tablets containing 900 mg total hypericin	3 tablets in the evening; 5 weeks	A randomized, double-blind, placebo-controlled, and cross-over study; 54 patients with painful polyneuropathy (up to 20 years)	Not changed significantly in individual pain ratings by SJW compared with placebo No significant effect on pain in polyneuropathy	(42)
PSD	Shugan Jieyu^7 ^capsule	0.72 g BID; 8 weeks	Clinical trials; 15 right-handed PSD patients (50-70 years)	↓ Depressive symptoms assessed by HAMD-24 significantly ↑ Cognitive functions assessed by MoCA ↑ Cognitive function through alteration of brain dynamics	(49)
PSD	Shugan Jieyu capsule	720 mg TID; 90 days	A RCT; 254 patients with acute ischemic stroke (up to 18 years)	Use of Shugan Jieyu yielded similar improvements in motor recovery after ischemic stroke compared with fluoxetine	(50)
Relapse in mild to moderate depression	STW3-VI^8^	900 mg QD; 6 weeks	A double-blind RCT; 154 patients with mild to moderate depression (18–74 years)	↓ Relapse rate compared with the placebo group and citalopram-treated patients No difference in the severity of relapse ↑ Duration of response compared with other groups	(60)
Relapse of depression	WS® 5570^9^	300 mg TID; 6 weeks acute, 26 weeks continuation, and 52 weeks maintenance treatment	A double-blind long-term RCT; 426 patients with a recurrent episode of major depression (18–65 years)	↓ Relapse rates during continuation treatment (18.1%) compared with placebo (25.7%) Beneficial effect in preventing relapse after recovery from acute depression Equal tolerability compared with placebo	(61)
Short-term memory	Remotiv^10^	500 mg or 250 mg; Single dose	A single dosage double-blind RCT; 82 student participants (51 males and 33 females)	↑ Mood in both dosages A significant positive effect of Remotiv 250 on digit span Negative effect of Remotiv 500 on digit span	(62)
Social anxiety disorder	L160	Flexible dose (300 -1800 mg) with minimum dose of 300 mg BID; 12 weeks	A randomized, double-blind, placebo-controlled trial; 40 patients with a primary diagnosis of Social Phobia (18–65 years)	No significant difference in Leibowitz Social Anxiety Scale between SJW and placebo	(43)
Somatoform disorders	LI 160	300 mg BID; 6 weeks	A randomized, double-blind, and placebo-controlled trial; 184 outpatients with somatization disorder (18-65 years)	45.4% of patients responded to SJW compared with 20.9% to placebo Tolerability of SJW treatment was equivalent to placebo	(63)

^1 ^LI 160 is a hydroalcoholic dried extract that has been standardized to total hypericin content (0.12–0.28%) and a range of analytical marker substances; ^2 ^Multi-fractionated extract with 0.3 % hypericin; ^3 ^Mono-fractionated extract with 0.3 % hypericin; ^4 ^Helarium-425® is 425 mg dry ethanolic extract in capsule that is standardized to hypericin 0.1–0.3% and 6% of hyperforin; ^5 ^Tablet containing 100 mg of SJW dried extract (L1160) standardized to 0.28% hypericin; ^6 ^Extract contains 60 mg SJW, 28 mg Valeriana officinalis, and 32 mg Passiflora incarnata L.; ^7 ^It is a Chinese herbal medicine mainly composed of Acanthopanax and SJW; ^8^ STW 3-VI is a extract from SJW (extraction solvent ethanol 80%,v/v); ^9 ^WS_ 5570 is a methanol extract from Herba hypericin, main constituents of which include 3–6% hyperforin, 0.1–0.3% hypericin, not less than 6% flavonoids; ^10^ A film-coated tablet of Ze 117 containing either 500 mg (Remotiv 500) or 250 mg (Remotiv 250) of dry extract from SJW that contains 0.1–0.3% of total hypericin

SJW: St John’s wort

**Table 2:** Clinical trials of St. John’s wort on endocrine disorders

**Disease/Activity**	**Extract/Compd.**	**Dosing & Duration**	**Study Design & Sample Size**	**Result**	**Ref.**
Androgenic steroid hormones	Kira®	300 mg TID; 2 weeks	An open-label crossover study; 12 volunteers (22–38 years) with a normal weight (72.9 ± 19.1 kg)	↓ 5α-reduced steroids levels ↑ Testosterone to DHT ratio	(69)
Endocrinological effects	WS® 5570	Several dosages (600, 900, and 1,200 mg); 4 different days	A single-blind study; 12 healthy male volunteers (26–41 years)	↑ ACTH No change in cortisol and prolactin	(70)
Menopausal symptoms	Perforan^®^^1^	One tablet TID; 8 weeks	A double-blind RCT; 80 postmenopausal women (45–60 years)	↓ Frequency and intensity of hot flashes ↓ Score of the Kupperman scale significantly ↓ Intensity of depression significantly	(64)
Menopausal symptoms	Tablet of SJW^2^	3 tablets TID; 16 weeks	A double-blind parallel RCT; 100 postmenopausal women (40–60 years)	No significant differences for daily weighted flushes or scores No significant change in the quality of life	(71)
Menopausal symptoms	Effervescent tablet of SJW and Pass P® drop of Passion Flower	160 mg effervescent tablet TID and 10 drops TID and 20 drops before sleep; 6 weeks	Clinical-experimental; 59 women who were in their first 5-year period of menopause	↓ Average score of menopause symptoms significantly	(72)
PCOS	Tablet 1: a combination of *Glycyrrhiza glabra*, *Paeonia lactiflora*, and Cinnamomum Tablet 2: *Tribulus terrestris* extract equivalent to 13.5 g aerial parts	Three tablet QD; Tablet 2: three tablets QD for 10 consecutive days commenced on menstrual cycle day 5 for oligomenorrhoeic women and within 1 week of trial commencement for women with amenorrhoea; 12 weeks	RCT; 122 women with PCOS (45–55 years)	Significant improvements in BMI, blood pressure, insulin, LH, anxiety, quality of life, stress, depression, and pregnancy rates	(68)
PMS	LI160	Tablets 900 mg QD; two menstrual cycles	A double-blind crossover RCT; 36 patients (18–45 years) with regular menstrual cycles with mild PMS	↑ Physical and behavioral PMS symptoms improvement No significant effects compared with placebo for mood- and pain-related PMS symptoms	(65)
PMS	Pills of SJW^3^	600 mg QD; 6 weeks	A double-blind RCT; 51 single women	No significant differences in BDI, VAS, or total PAF ↑ Emotional lability, hostility/anger, and impulsivity scores significantly	(73)
PMS	680-μg hypericin	2 tablets QD; 8 weeks	A double-blind RCT; 170 women with PMS (for at least 6 months)	↓ PMS scores compared with baseline and the control groups ↓ Crying (71%) and depression (52%) scores	(66)
Premenopausal syndrome	Ethanol extract	900 mg TID; 12 weeks	A pilot double-blind, randomized trial; 106 premenopausal women of whom 47 completed the study (40-65 years)	↑ Menopause-specific quality of life ↓ Sleep problems Non-significant difference in the daily hot flash frequency	(74)
Premenopausal syndrome	Hypiran^®^ drop^4^	20 drops TID; 8 weeks	A double-blind RCT; 100 women experiencing hot flashes (45-55 years)	↓ Severity of flashes	(67)

^1 ^A tablet from an Iranian drug company that contains 270–330 μg of SJW; ^2^ Each tablet contained 300 mg extract equivalent to 1,800 mg dry herb flowering top standardized to contain 990 µg of hypericin, 9 mg of hyperforin, and 18 mg of flavonoid glycosides; ^3^ Each pill contained 0.3% hypericin and 3% hyperforin; ^4^ The St John’s wort drops contained 0.2 mg/ml hypericin

SJW: St John’s wort

**Table 3:** Clinical trials of St. John’s wort on skin diseases

**Disease**	**Extract/Compd.**	**Dosing & duration**	**Study design & sample size**	**Result**	**Ref.**
BMS	Extract (hypericin 0.31% and hyperforin 3.0%)	300 mg TDS; 12 weeks	A double-blind, single-center RCT; 39 patients (mean age of 64.9 ± 4.7 years)	No decrease in pain of BMS significantly	(80)
Episiotomy wounds	Ointments of *Achillea millefolium* and SJW^2^	Rubbing 1 cm of the ointment on the area of episiotomy BID; 10 days	A double-blind clinical trial; 140 primiparous women (37–42 years)	↓ Pain level, ↓ Redness edema ↓ Ecchymosis	(75)
HSV-1 and HSV-2 lesions	Dynamiclear™^3^	Dynamiclear QD; 2 weeks	A prospective, randomized, multi-centered, comparative, open-label trial; 149 patients (18–55 years) with active HSV-1 and HSV-2 lesions	↓ Burning sensation and parameters of acute pain, erythema, and vesiculation compared with acyclovir No significant adverse effects and well tolerated	(77)
Non-melanoma skin cancer	Topical application with hypericin^4^	Topical use on the lesions, 10 mm of surrounding skin in a 1 mm thick layer under occlusive dressing; 6 weeks	A pilot study; 34 patients: 8 with AKs, 21 with basal cell carcinoma and 5 with Bowen’s disease (32–83 years)	↑ Percentage of complete clinical response Partial remission in patients with nodular BCCs A complete disappearance of tumor cells	(81)
Plaque-type psoriasis	Ointment: SJW (5% w/w), vaseline (84% w/w), propylene glycol (10% w/w) and avicel (1% w/w)	Ointment BID; 4 weeks	A pilot single-blind study; 10 patients (20–55 years) with mild plaque psoriasis	↓ PASI significantly ↓ Erythema, scaling, and thickness	(78)
Plaque-type psoriasis	Ointment^5^: SJW (5% w/w), vaseline (84% w/w), propylene glycol (10% w/w), and avicel (1% w/w)]	Using ointment BID; 4 weeks	A double-blind, placebo-controlled, pilot study; 20 patients with mild to moderate plaque-type psoriasis on both sides of the body (18–55 years)	↓ Erythema, scaling, and thickness significantly ↓ TNFα concentrations in the dermis, endothelial cells, and dendrite cells significantly	(79)
Scar of cesarean	Ointment^1^	Ointment TID; 16 days	A double-blind RCT; 144 women with surgical childbirth (17–35 years)	↓ Pain and pruritus significantly	(76)

^1 ^The hydro alcoholic condensate extracts packed with sterile Vaseline as the base (%5 weight ratio) in 30 gr tubes; ^2 ^Topical formulation containing SJW, Calendula Officinalis, and copper sulfate; ^3 ^The products contained pseudohypericin (67.5%) and hypericin (32.5%); ^4 ^It was prepared from an extract of SJW (5% w/w), vaseline (84% w/w), propylene glycol (10% w/w), and avicel (1% w/w); ^5^ Oily extract provided by the Gol-Daru Company (Isfahan, Iran)

SJW: St John’s wort

**Table 4:** Clinical trials of St. John’s wort co-administered with other drugs

**Interaction/Co. administration**	**Extract/Compd.**	**Dosing & duration**	**Study design & sample size**	**Result**	**Ref.**
Ambrisentan	Jarsin^®^	300 mg TID; 10 days (tenth to twentieth day of study)	An open-label, monocentric, one-sequence, crossover, multiple-dose clinical trial; 20 healthy volunteers (10 CYP2C19 extensive, 4 poor, and 6 ultra-rapid metabolizers) (mean age of 31.3 ± 7.7 years)	Equality of ambrisentan concentration in extensive, ultra-fast, and poor metabolizers ↓ Ambrisentan exposure (17–26%) in all genotype groups No significant reduction in the effect of CYP2C19 on the metabolism of ambrisentan	(89)
Atorvastatin	Movina^®^	300 mg BID; 12 weeks	An open, crossover RCT; 16 patients with hypercholesterolemia (55-72 years)	↑ LDL cholesterol serum level significantly ↑ Total cholesterol No statistically significant change in HDL cholesterol and triglycerides	(88)
Boceprevir	Ucalm^®^^1^	2 tablets QD; 56 days (SJW on days 1–14, SJW plus boceprevir (SJW on days 22–35 and together on days 31–35) and boceprevir on days 52–56, separated by 7-day washout periods)	Phase I, open-label, three-period, cross-over trial; 17 healthy subjects (26–49 years)	Failure to observe clinical effects on the plasma concentration of boceprevir (or its metabolite)	(121)
Bupropion	SJW extract	325 mg TDS; 2 weeks	An open-label, two-phase design; 18 healthy males	↑ Oral clearance of bupropion ↓ Area under the concentration versus time curve extrapolated to infinity of bupropion	(93)
Carbamazepine	Extract standardized to 0.3% hypericin	300 mg TID; 2 weeks	An Open-label Trial; 8 healthy subjects	No change in Cmax and AUC of carbamazepine	(122)
Cyclosporin A	Jarsin^®^	600 mg QD; 2 weeks	An open-label study; 11 renal transplant patients (34–59 years)	↓ AUC_0-12_, C _max_ and C _trough_ values for cyclosporin significantly by 46% ↓ Plasma cyclosporin concentrations	(94)
Cyclosporine	Jarsin^®^ with low (0.1 mg) and high (7.0 mg) concentrations of hyperforin	(900 mg/d) containing low or high concentrations of hyperforin; 2 weeks	A crossover study; 10 renal transplant patients (25–65 years)	↓ Plasma ciclosporine levels significantly Not influence cyclosporine pharmacokinetics significantly by extract with low hyperforin content	(85)
Cytochrome P450 enzymes and P-glycoprotein	Rebalance®^2^ 500	500 mg QD; 10 days	An open-label, non-randomized, single-sequence study; 20 healthy volunteers (18-55 years)	No pharmacokinetic interactions of Ze 117 for CYPs and P-glycoprotein No relevant pharmacokinetic interactions with important CYPs and P-glycoprotein	(86)
Digoxin	variable formulation of SJW	Variable concentration of hyperforin; 2 weeks	A parallel-group RCT; 96 healthy volunteers (18-40 years)	No significant interaction with 2 g powder without hyperforin, tea, juice, oil extract, hyperforin-free extract (Ze 117), or low daily doses of hyperforin-containing *Hypericum *powder (1 g, 0.5 g) and placebo ↓ AUC_0-24_, C_max_, and C_trough_ of digoxin with high-dose hyperforin-rich extract (LI 160)	(87)
Docetaxel	Hyperiplant®^3^	300 mg TDS; 2 weeks	An open-label, non-randomized, crossover study; 10 patients with histological or cytological proof of cancer for whom treatment with docetaxel (up to 18 years)	↓ Mean area under the docetaxel plasma concentration-time curve significantly ↑ Docetaxel clearance significantly ↓ Incidence of docetaxel-related toxicities	(95)
Effect of macitentan before and during SJW on the pharmacokinetics of rivaroxaban	Jarsin®	300 mg TID; 12 days	An open-label, monocentric, two-period, one sequence phase I clinical trial; 12 healthy volunteers (up to 18 years)	↑ CYP3A activity by 272% ↓ GMR of rivaroxaban AUC and Cmax by 25% ↓ GMR of macitentan AUC by 48% and of C_max_ by 45%	(99)
Effect of SJW and ritonavir on Cyp3A enzyme activity	Jarsin^®^	300 mg TDS; 2 weeks	An open, fixed-sequence study design; 12 healthy Caucasian participants (mean age of 26±3.25 years)	↑ (AUC)_0–8 h_ of midazolam	(111)
Effect of SJW on CYP2C19 activity	Extract with 4% hyperforin and 0.3% hypericin	300 mg TDS; 2 weeks	A two-phase, randomized, crossover design; 12 healthy males (6 extensive metabolizers of CYP2C19 and 6 poor metabolizers) (18–25 years)	↑ CYP2C19 activity significantly No significant alteration in CYP2C19 poor metabolizers	(106)
Effects of SJW and ketoconazole (CYP3A inhibitor) on CYP3A	Jarsin^®^	300 mg TDS; 8 days	A two-phase, randomized, cross-over, open, monocentral trial; Twelve healthy, male participants (22–49 years)	↓ Clearance of midazolam in relation to baseline (82%) strongly by a single dose of ketoconazole when used concomitantly with SJW	(110)
Fluoxetine	LI-160	300 mg TDS; 12 weeks	An active, parallel-group, double-blind RCT; 134 patients with MDD (mean age of 37.3 ± 11.0)	↓ HAMD-17 scores at the endpoint in the SJW group ↑ Remission rates (HAMD-17 <8)	(109)
Ibuprofen	Extract standardized with 0.3% hypericin	300 mg TID; 3 weeks	An open-label trial; 8 male subjects	No change in Cmax and AUC of ibuprofen	(123)
Imatinib	Kira®	300 mg TID; 17 days	An open-label trial;12 healthy subjects (20–51 years)	↓ Cmax, AUC, and t1/2 ↑ Clearance of imatinib	(115)
Indinavir	Extract standardized with 0.3% hypericin	300 mg TID; 2 weeks	An open-label study; 8 healthy males	↓ Cmax and AUC	(112)
Intravenous fentanyl	Extract Kira®	300 mg TID; 20 days	A randomized parallel-group design; 16 healthy subjects (21–41 years)	No effect on fentanyl pharmacokinetics, pharmacodynamics, or clinical effects No influence on analgesia, cognitive performance, or somatic cognitive–affective effects of fentanyl	(90)
Irinotecan	SJW extract (300 mg)^4^	300 mg TDS; 18 days	An unblinded, randomized crossover study;5 cancer patients	↓ Plasma levels of SN-38 (active metabolite of irinotecan)	(101)
Ivabradine	Jarsin^®^	300 mg TID; 2 weeks	A non-randomized, open-label trial; 18 healthy subjects (18-40 years)	↓ C max and AUC of ivabradine and its active metabolite	(124)
Metformin	Modigen®^5^	One capsule BID; 3 weeks	An open cross-over study; 20 healthy male subjects (18–64 years) who received 1 g of metformin twice daily for 1 week	↓ Renal clearance of metformin ↓ Area under the glucose concentration–time curve ↑ Glucose tolerance by enhancing insulin secretion independently of insulin sensitivity	(102)
Midazolam	Capsule with low hyperforin (total hyperforin 0.06 ± 0.001 mg and total hypericin 0.60 ±0.03 mg)	500 mg BID; 2 weeks	An open-label one-sequence crossover, single-dose study; 20 healthy male volunteers (mean age of 24.9 ± 2.3 years)	↓ Midazolam AUC0–∞ slightly No significant change in C_max_, t_1/2_ and t_max_ of midazolam Mild induction of CYP3A	(91)
Oral contraceptives	SJW extract containing 0.3% hypericin and 20 ng/ml average steady-state concentrations of hyperforin	300 mg TDS; for 3 consecutive 28-day menstrual cycles	Clinical trials; 12 healthy premenopausal women (mean age of 27 ± 7 years)	↑ Oral clearance of norethindrone ↓ Half-life of ethinyl estradiol significantly ↑ CYP3A activity	(96)
Oral contraceptives	Extract with 0.3% hypericin and 3.7% hyperforin	300 mg TDS; 4 consecutive 28-day cycles	A single-blind sequential trial; 16 healthy women	↓ Dose exposure from the contraceptive significantly by 13–15% ↑ Breakthrough bleeding and follicle growth and ovulation	(103)
Oral oxycodone	Jarsin^®^	300 mg TID; 2 weeks	A cross-over RCT; 12 healthy volunteers (mean age of 23 ± 4 years)	↓ AUC of oxycodone by 50% ↓ The plasma concentrations of oral oxycodone	(97)
Oral S-ketamine	Jarsin^®^	300 mg TID; 2 weeks	A cross-over RCT; 12 healthy subjects (20–35 years)	↓ (AUC0–∞) of ketamine by 58% ↓ Cmax of ketamine by 66% No significant changes in the behavioral or analgesic effects of ketamine	(104)
Paroxetine	WS® 5570	900 mg/day (initially non-responders’ doses were increased to 1800 mg/day); 6 weeks	A double-blind, double-dummy, reference controlled, multicenter non-inferiority RCT; 251 adult outpatients with acute major depression (18-70 years)	↓ Hamilton depression total score ↓ Incidence of adverse events	(108)
Platelet response in patients resistant to clopidogrel after PCI	SJW extract	300 mg TDS; 2 weeks after PCI	A single-center 2:1 open-label RCT; 23 patients’ non-responders to 600 mg clopidogrel (18–75 years)	↑ Residual platelet reactivity during the first-month post-PCI Changed PRU significantly	(84)
Prednisone	Extract standardized with hypericin 0.3%	300 mg (tablets) TID; 4 weeks	A single-dose study; 8 healthy males (19–36 years)	No significant alterations in the pharmacokinetic parameters for prednisone or prednisolone	(92)
Repaglinide	SJW extract	325 mg TID; 14 days	A two-phase, randomized, crossover study; 15 healthy subjects with specific solute carrier organic anion transporter family member 1B1 (SLCO1B1) Genotypes (19–24 years)	No effect on the total area under the plasma concentration-time curve from time zero to infinity (AUC∞), the elimination half-life (t½), or the peak plasma concentration (C_max_) No significant effect on the blood glucose-lowering and insulin-elevating effects of repaglinide	(125)
Response of clopidogrel in hypo-responsive volunteers	Kira^®^	300 mg TID; 2 weeks	A prospective, randomized, double-blind, pilot study; 10 healthy clopidogrel hypo responsive volunteers (18–70 years)	↓ Platelet aggregation ↑ CYP3A4 activity	(107)
Rifampicin	Jarsin®	Flexible dose (300–600 mg TID) (first 300 mg QD for 14 days, second 300 mg TID for 14 days and finally increased to 600 mg TID within 3–6 days	Clinical phase I trial; 12 healthy volunteers (six males and six females)	↑ Dermatological and neurological symptoms in sun-exposed areas only in women	(98)
Rosuvastatin	Capsule including 300 mg SJW 80 mg rosemary, and 40 mg spirulina	300 mg BID; 20 weeks	Case report; one male with hypercholesterolemia	↑ LDL-cholesterol ↑ Total-cholesterol	(126)
Simvastatin	Movina^®^^6^	300 mg BID; 4 weeks	A controlled, randomized, open, crossover study; 24 patients with hypercholesterolemia (54–78 years)	↑ LDL-cholesterol significantly ↑ Total-cholesterol	(88)
Tacrolimus	Jarsin^®^	300 mg TDS; 18 days	A clinical trial;10 healthy volunteers (20–30 years)	↓ AUC of tacrolimus significantly ↑ Oral clearance and oral volume of distribution at steady state of tacrolimus	(100)
Theophylline	TruNature® with 0.3% hypericin	300 mg TDS; 15 days	A randomized, open-labeled, crossover study; 12 healthy Japanese male volunteers (mean age of 25.0 ±6.4 years)	No significant changes in the pharmacokinetics of theophylline in plasma	(127)
Warfarin	Tablets contain SJW with 12.5 mg hyperforin and 0.825 mg hypericin and Korean ginseng	One tablet TID; 3 weeks	An open-label, crossover randomized trial; 12 healthy males (20–40 years)	↓ AUC and t1/2 ↑ Clearance of S-warfarin and R-warfarin	(120)
Zolpidem	LI160	300 mg TDS; 2 weeks	A controlled, open-label, non-randomized, fixed-dose schedule design; 14 healthy males (mean age of 21.1 ±1.5 years)	↓ Zolpidem plasma concentration by enhancing CYP3A4 activity	(105)

^1 ^It includes 300 mg of SJW extract per tablet; ^2 ^SJW dry extract Ze 117 contained hyperforin (0.96 mg) per film-coated tablet; ^3^ Dry extract standardized to 0.36–0.84 mg hypericin and 9–19 mg hyperforin; ^4^ Bio Nutrition Health Products, Den Bosch, The Netherlands; ^5^ Capsule with 240–294 mg dry extract of SJW (900 μg total hypericin); ^6^ Capsules contained 300 mg SJW extract standardized to 3-6 % hyperforin

SJW: St John’s wort

## Conclusion

*Hypericum perforatum*, known as Saint John’s wort, is a medicinal plant widely used for psychiatric problems. Hence, most of the clinical trials performed on SJW are related to psychological problems. However, according to many *in vitro*, *in vivo, *and clinical studies it has promising effects for a range of disorders including infectious problems and skin disorders that could replace routine treatments by physicians in the future. One of the most important features of this plant is the variety of drug interactions that it can cause. The results of this article can be a guide for researchers to design stronger and more complete studies in the future.

## Authors’ Contributions

SZN Helped with writing the original draft, investigation, and methodology; MA Provided data curation, writing, review, editing, and formal analysis; AM and ATM Helped write, review, and edit; SAE Provided supervision and helped write, review, and edit.

## Funding Source

This work did not receive any financial support.

## Conflicts of Interest

The authors have no conflicts of interest to declare.
